# Cytomorphological characteristics of glassy cell carcinoma of the uterine cervix: histopathological correlation and human papillomavirus genotyping

**DOI:** 10.18632/oncotarget.12361

**Published:** 2016-09-30

**Authors:** Yoon Yang Jung, Ji Hae Nahm, Hyun-Soo Kim

**Affiliations:** ^1^ Department of Pathology, Myongji Hospital, Seonam University College of Medicine, Goyang-si, Republic of Korea; ^2^ Department of Pathology, Asan Medical Center, University of Ulsan College of Medicine, Seoul, Republic of Korea; ^3^ Department of Pathology, Severance Hospital, Yonsei University College of Medicine, Seoul, Republic of Korea

**Keywords:** cervix, glassy cell carcinoma, cytology, human papillomavirus

## Abstract

A retrospective analysis was performed to describe the cytomorphological and histopathological findings and human papillomavirus (HPV) genotypes for glassy cell carcinoma (GCC) of the uterine cervix. Five cases of cervical GCC, in which the glassy cell features constituted at least 95% of the specimen, were included. Four patients had stage IIB GCCs and one had stage IIIB GCC. All patients underwent concurrent chemoradiation therapy. Based on pretreatment cytology, only 1 of the 5 cases was correctly diagnosed as GCC. The remaining cases were diagnosed as carcinoma of undetermined type, adenocarcinoma, poorly differentiated carcinoma, or unsatisfactory for evaluation. Cytological specimens had moderate cellularity and contained small clusters of tumor cells admixed with amphophilic, granular tumor diathesis. The tumor cells possessed large, round to oval nuclei and abundant, granular, ground-glass cytoplasm. The nuclei exhibited prominent eosinophilic nucleoli. The cytoplasm displayed sharp margins and molding, resulting in “intercellular windows” between neighboring attached cells. HPV genotyping revealed that high-risk HPV types 18, 16, and 31 were detected in 3, 1, and 1 cases, respectively. Consistent with this finding, all cases exhibited block p16 positivity, confirming the association of HPV infection with GCC. In conclusion, a distinct cytoplasmic margin, the characteristic histopathological feature of GCC, was observed in liquid-based cytological preparations. We suggest that sharp cytoplasmic outlines with molding and intercellular windows are characteristic cytomorphological features of GCC. Detection of high-risk HPV in all cases strongly supported the notion that high-risk HPV is involved in the pathogenesis of GCC.

## INTRODUCTION

Glassy cell carcinoma (GCC) of the uterine cervix is a rare pathological form of cervical carcinoma that occurs in 1-2% of all cases [1–3]. The World Health Organization Classification currently considers this tumor to be a subtype of “other epithelial tumors” [4]. The mean patient age of those with GCC is ~10 years less than that of patients with squamous cell carcinoma or adenocarcinoma of the cervix [1, 2, 5]. GCC was first described by Cherry and Glucksmann [6] in 1956 as a specific and distinctive entity of the most poorly differentiated adenosquamous carcinoma. They defined the histological diagnostic criteria and indicated that this tumor was uncommon and associated with poor prognosis. Two decades later, Littman et al. [2] described GCC tumors in detail, redefining and amplifying the histological criteria. Since then, interest in GCC has expanded and a number of studies have been conducted [1–3, 7–22]. However, cervical GCC is not yet widely recognized. Furthermore, because of GCC rarity there have been no clinical trials or large cohort studies.

Early detection of cervical cancer greatly increases the successful treatment rate. In countries with established early detection programs, the impact of cytology-based cervical screening is reflected in the substantial reduction in the cervical cancer incidence over the past 50 years [23–27]. Although the aggressive clinical course of GCC encourages the use of early detection methods because data on the cytological features of GCC are limited, a cytological diagnosis can be difficult, despite characteristic histopathological features [1–4, 17]. Liquid-based cytology can improve specimen quality by providing a standardized method of collecting cervicovaginal material, and dispersing cells in a thin layer that is relatively inflammation-free [28–30]. This reduces the likelihood of unsatisfactory smears and increases the cytomorphological abnormality detection rate [31–37]. Although liquid-based cytology has become a common screening method for cervical cancer, limited information is available on the liquid-based cytology findings of GCC.

The aim of the present study was to describe the cytomorphological and histopathological features and immunophenotypes of cervical GCC. In addition, human papillomavirus (HPV) infection status in cervical GCCs was evaluated. We found characteristic cytomorphological features, and verified that those are consistent with typical histopathological features of GCC. We observed high-risk HPV infection and block p16 positivity in all examined cases.

## RESULTS

### Patient demographics

During the period from July 2007 to June 2016, 675 patients were diagnosed as having primary cervical carcinoma. Thirty-three (4.9%) cases of poorly differentiated carcinoma were collected from computerized patient records. Based on the rarity of GCC, we reviewed the 9 (1.3%) cases of cervical poorly differentiated adenosquamous carcinoma. Among the 9 cases, 2 cases that presented glassy cell features but that also had squamous (1 case) or glandular (1 case) differentiation, and 2 cases that had diffuse, strong p40 expression were excluded. The remaining 5 (0.7%) cases were cervical GCCs. Five patients with cervical GCC were included in a retrospective analysis. The median and mean patients ages at diagnosis were 38 and 48.2 years, respectively (range, 36-67 years). Four (80.0%) of the 5 patients had International Federation of Gynecology and Obstetrics (FIGO) stage IIB GCCs and the remaining 1 (20.0%) had FIGO stage IIIB GCC. None of the patients were pregnant or had a recent history of pregnancy. All patients underwent concurrent chemoradiation therapy.

### Cytological findings

**Figure 1:**
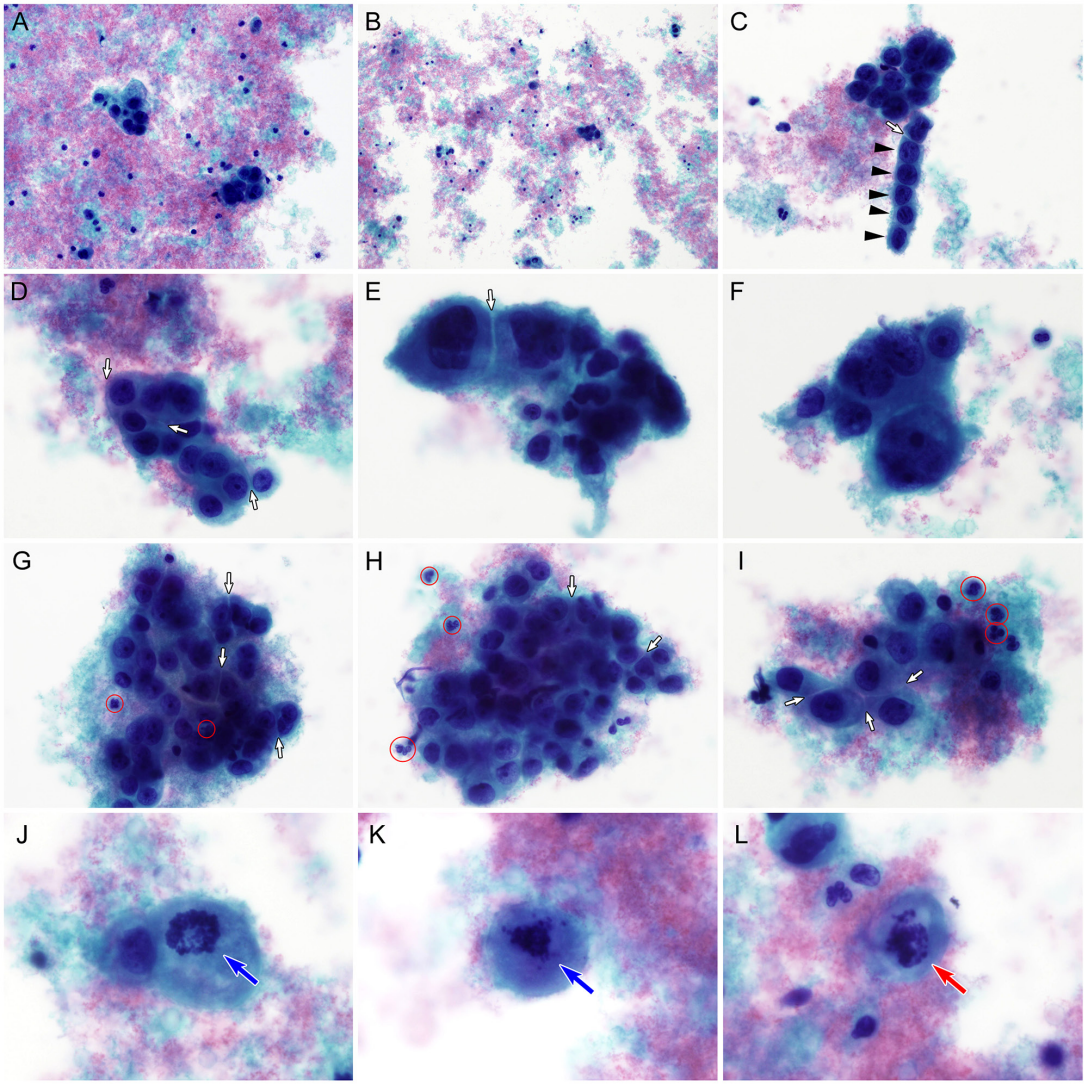
Liquid-based cytological findings of cervical glassy cell carcinoma **A.** Thinprep preparation of the cervical sample shows amphophilic, granular necrotic debris (tumor diathesis) and a few small clusters of polygonal tumor cells. **B.** The tumor cell clusters or individually scattered tumor cells are unevenly distributed. Tumor diathesis is apparent. **C.** Although some tumor cells display endocervical-like pseudocolumnar arrangements (black arrowheads), there is no definite evidence of glandular differentiation. A white arrow indicates the intercellular window. **D.** The tumor cells have relatively fine chromatin and prominent, solitary nucleoli. Abundant, cyanophilic cytoplasm and discrete cell borders (white arrows) are evident. **E.** Under high-power magnification (×400), the tumor cells show large, oval to round, pleomorphic nuclei and “intercellular windows” produced by discrete cytoplasmic outlines and cytoplasmic molding (white arrow). There are no intercellular bridges. **F.** Tumor cells are 3–7 fold larger than lymphocytes or neutrophils. Chromatin distribution irregularities, hyperchromasia, and significant anisonucleosis are apparent. **G-I.** In several areas, an intimate admixture of neutrophils (red circles) and tumor cells, so-called granuloepithelial complexes, is seen. Cytoplasmic molding and intercellular windows (white arrows) are observed. **J-K.** Mitotic figures (blue arrows) are present. **L.** Atypical mitotic figures (red arrow) are also detected (A-L, Papanicolaou stain).

The clinicopathological characteristics of the 5 patients with cervical GCC are summarized in Table 1. Based on pretreatment cytology, only 1 (20.0%) of the 5 cases was correctly diagnosed as GCC (case 4), although for the case with an unsatisfactory cytological specimen on the first examination, a cytological diagnosis of GCC was obtained from the recurrent tumor (case 3). The remaining cases were diagnosed as carcinoma, type undetermined (case 1), adenocarcinoma (case 2), or poorly differentiated carcinoma (case 5). There were no false negative results. Liquid-based cytology specimens displayed moderate cellularity and contained small clusters of polygonal tumor cells admixed with amphophilic, granular necrotic debris (tumor diathesis; Figure 1A). Most cellular clusters had < 20 cells. Even though most tumor cells were cohesive, individual tumor cells were frequently observed. A background of tumor diathesis was common (Figure 1B). Some tumor cells assumed a pseudocolumnar shape, resembling endocervical cells (Figure 1C), but there was no definitive evidence of glandular differentiation. Rarely, cellular clusters showing vague acinar architecture and nuclear polarization were noted, but insufficient for diagnosis of adenocarcinoma. There was no evidence of dyskeratosis or intercellular bridge formation. The tumor cells possessed large, round to oval nuclei with a thin, irregular nuclear membrane. Although most tumor cells had finely dispersed chromatin, some had coarse chromatin and thick nuclear membranes with dense parachromatin. A moderate to large amount of tumor cell cytoplasm stained faintly blue with Papanicolaou stain, giving a fine ground-glass appearance (Figure 1D). The cytoplasm displayed distinct outlines with molding and clear, slit-like spaces between neighboring attached cells (“intercellular windows”; Figure 1E). The tumor cells were 3–7 fold larger than lymphocytes, and the majority of their nuclei contained prominent nucleoli (Figure 1F). Occasionally, tumor cell phagocytosis of apoptotic neutrophils was observed. There was a mixed population of inflammatory cells, mainly plasma cells and lymphocytes, in the background. Eosinophils, though present, were not easily detected. An intimate admixture of neutrophils and tumor cells, a so-called granuloepithelial complex [13], was often identified (Figures 1G-1I). Cytoplasmic molding and intercellular windows were often seen in these complexes. Frequent mitoses (Figures 1J-1K) and atypical mitotic figures (Figures 1L) were detected.

**Table 1:** Results of cytology, biopsy, immunostaining, and HPV genotyping and treatment of cervical GCC

Case	Age	FIGO stage	Cytology result	Biopsy result	Immunostaining result					HPV genotype	Treatment
					p40	CEA	ER	PgR	p16		
1	38	IIB	Carcinoma, type undetermined	GCC	Negative	Negative	Negative	Negative	Block positive	HR HPV (type 18) detected	CCRT
2	63	IIB	Adenocarcinoma	GCC	Negative	Negative	Negative	Negative	Block positive	HR HPV (type 16) detected	CCRT
3	36	IIB	Unsatisfactory/GCC*	GCC	Negative	Negative	Negative	Negative	Block positive	HR HPV (type 31) detected	CCRT
4	67	IIIB	GCC	GCC	Negative	Negative	Negative	Negative	Block positive	HR HPV (type 18) detected	CCRT
5	37	IIB	Poorly differentiated carcinoma	GCC	Negative	Negative	Focal positive	Focal positive	Block positive	HR HPV (type 18) detected	CCRT

**Abbreviations:** CCRT: concurrent chemoradiation therapy, CEA: carcinoembryonic antigen, ER: estrogen receptor; FIGO: International Federation of Gynecology and Obstetrics, GCC: glassy cell carcinoma, HPV: human papillomavirus, HR: high-risk, PgR: progesterone receptor;

* At the time of recurrence

### Histopathological and immunohistochemical findings

**Figure 2:**
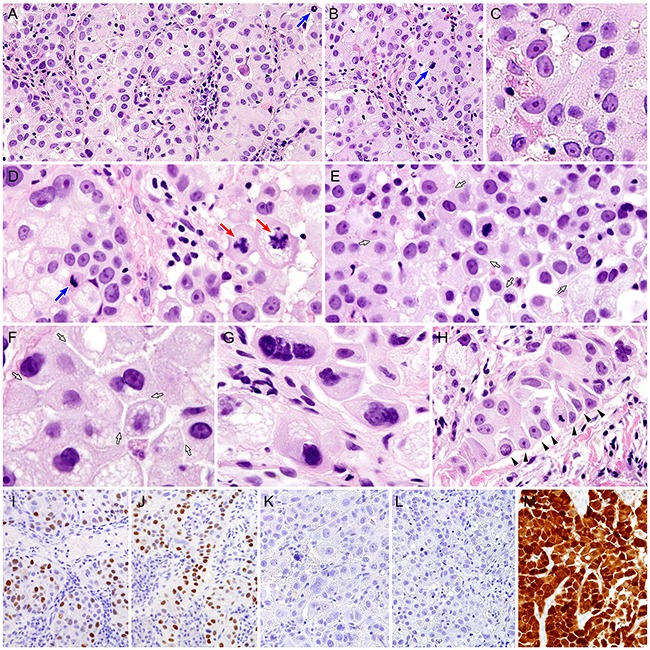
Histopathological and immunohistochemical findings of cervical glassy cell carcinoma **A.** Glassy cell carcinoma consists of tumor cell nests with pale to eosinophilic, abundant cytoplasm. Mitotic figures are frequently observed (blue arrow). **B.** The tumor cell nests are surrounded by thin fibrovascular connective tissue septa with lymphoplasmacytic infiltrate. A blue arrow indicates a mitotic figure within the tumor cell. **C.** The individual tumor cells display large nuclei with single, prominent nucleoli. **D.** Tumor cell cytoplasm possesses eosinophilic, granular cytoplasm, and some exhibit variable-sized microvacuoles. In several areas, mitotic figures are frequent (blue arrow), including abnormal forms (red arrows). **E.** The tumor cells show distinct cytoplasmic margins and “intercellular windows” (white arrows), which are also apparent in cytological specimens. **F.** Under high-power magnification (×400), cytoplasmic molding and clear, slit-like intercellular spaces (white arrows) are obviously observed, but intercellular bridging is absent. **G.** Some bizarre multinucleated giant cells are noted. **H.** Although cells with a pseudocolumnar arrangement can be seen (black arrowheads), there is no definite evidence of gland formation. **I-M.** Immunohistochemically, some tumor cells are positive for **(I)** estrogen receptor and **(J)** progesterone receptor in a single case. None of the cases examined shows immunoreactivity for **(K)** p40 or **(L)** carcinoembryonic antigen. In contrast, all cases exhibited block positivity for **(M)** p16 (A-H, hematoxylin and eosin stain; I-M, polymer method).

Histopathologically, the tumor tissue consisted of nests, cords, or sheets of large polygonal cells with a moderate to large amount of pale, evenly spread, finely granular or ground glass-appearing cytoplasm (Figure 2A). The tumor cell nests were separated by thin, delicate fibrovascular septa, containing a cellular infiltrate, which consisted predominantly of plasma cells and lymphocytes admixed with eosinophils (Figure 2B). The individual tumor cells exhibited sharp cytoplasmic outlines and large, pleomorphic nuclei with prominent nucleoli (Figure 2C). Mitotic figures were numerous and included abnormal forms (Figure 2D). Under high-power magnification (×400), the distinct cytoplasmic margins that were seen on the cytological specimens were often observed. Cell shape variation associated with cytoplasmic molding and intercellular windows was consistent with that found on the cytology (Figures 2E-2F). Some tumor cells displayed cytoplasmic microvacuolation. Bizarre multinucleated giant cells were commonly seen (Figure 2G). There was no evidence of squamous pearl formation, dyskeratosis, or intercellular bridge formation. Although a pseudocolumnar arrangement of the tumor cells that was observed on the cytological specimens was also found (Figure 2H), there was no evidence of gland formation or mucin production. A few microscopic foci of coagulative tumor cell necrosis were present.

Immunohistochemical staining results are shown in Table 1. Estrogen (ER; Figure 2I) and progesterone (PgR; Figure 2J) receptors were focally positive in 1 case. None of the cases showed p40 (Figure 2K) or carcinoembryonic antigen (CEA; Figure 2L) expression. In contrast, all cases exhibited diffuse and strong nuclear and cytoplasmic p16 immunoreactivity (block positivity; Figure 2M).

### HPV genotype status

HPV genotype was analyzed using HPV DNA chip assay. High-risk HPV was detected in all cases (Table 1). This result was in agreement with the block p16 positivity observed in all cases. The genotype was variable; type 18 was detected in three (60.0%) cases, and type 16 (20.0%) and type 31 (20.0%) was detected in 1 case for each. Multiple HPV genotypes were not observed.

## DISCUSSION

It has been documented that GCC does not have a distinctive cytological appearance [2, 3, 17]. In the present study, a pretreatment cytological diagnosis of GCC was made in only 1 case examined. Similar to our observation, in a previous study, 1 of 9 (11.1%) GCC cases were correctly diagnosed on a cervicovaginal smear [13]. The remaining cases were diagnosed as squamous cell carcinoma (3 cases), adenocarcinoma (1 case), adenosquamous carcinoma (1 case), or carcinoma, unknown type (3 cases). The reason for such a low diagnostic accuracy in cytology might be attributed to the lack of differentiation and a low suspicion index for GCC. However, pathologists should be aware of the cytomorphological features of GCC because it not only has relatively unique cytomorphology but also diagnostic pitfalls, leading to occasional misdiagnosis. In our series, the established histopathological findings of GCC were verified in the cytological specimens. Clusters of tumor cells with abundant, granular cytoplasm, pleomorphic nuclei, and prominent nucleoli were consistent with GCC histomorphology. In addition, sharp cytoplasmic outlines of GCC cells associated with cytoplasmic molding and intercellular window formation were apparent in liquid-based cytological preparations, and these were confirmed on histopathological examination. Although sharp cytoplasmic margins have been suggested as a histopathological characteristic of GCC, there have been no reports concerning cytoplasmic molding and intercellular windows as cytomorphological features. Since these 2 features are typically found in reactive or neoplastic mesothelial lesions, it is difficult to conclude that they are pathognomonic for cervical GCC. However, because cytoplasmic molding and intercellular windows are not found in liquid-based cytology of common cervical neoplastic lesions such as high-grade squamous intraepithelial lesion, squamous cell carcinoma, adenocarcinoma in situ, and endocervical adenocarcinoma, it could be inferred that at least cytoplasmic molding and distinct intercellular windows could be useful cytological features to diagnose GCC.

Previous studies have found a strong association between cervical GCC and high-risk HPV infection [3, 7–12], which would be consistent with the findings in the present study. According to an accumulation of the present and previous data, the overall prevalence of high-risk HPV infection would be 51.3% (20/39; Table 2). Interestingly, 3 previous cases showed multiple high- and low-risk HPV infections [12], whereas in the present study high-risk HPV alone was noted. In the present study, 3 (60.0%) cases had HPV type 18, which was the most common type detected in previous studies (10/15; 75.0%). HPV type 18 is most commonly associated with glandular tumors of the uterine cervix [4, 7, 8], which leads to the argument that GCC should be identified as a subtype of adenocarcinoma rather than of squamous cell carcinoma; however, the GCC glandular lineage cannot be proven because HPV type 18 is not specific to glandular lesions. In fact, HPV type 18 is the second most common HPV types in cervical squamous epithelial lesions and is strongly associated with high-grade neuroendocrine carcinomas [38]. Therefore, it is reasonable to assume that HPV type 18 has an ability to make a target cell differentiate in variable directions [8]. Although the pathogenetic importance of high-risk HPV infection in cervical GCC could not be determined definitively because of the small number of cases reported, based on the results of the present and previous studies there is a high possibility that it is involved in GCC carcinogenesis. Further studies are needed for a full evaluation of HPV infection status in GCC of the uterine cervix.

**Table 2:** Previously reported HPV prevalence rate and genotypes in cervical GCC

Year published	Author	HPV prevalence rate	HPV genotype		
			Category	Type (number of case)	Detection method
1998	Kenny et al. [7]	27.8% (5/18)	HR	18 (4), 16 (1)	ISH
2002	Kato et al. [8]	66.7% (2/3)	HR	18 (2)	PCR
2004	Hirai et al. [9]	100.0% (2/2)	HR	18 (2)	PCR
2004	Ng et al. [10]	0.0% (0/1)	Not detected		PCR-RFLP
2004	Matthews-Greer et al. [11]	100.0% (1/1)	HR	16 (1)	PCR
2009	Kim et al. [12]	55.6% (5/9)	HR and LR	18 (2), 31+32 (1),35+68+32 (1), 39+6 (1)	DNA chip
2016	Jung et al.*	100.0% (5/5)	HR	18 (3), 16 (1), 31 (1)	DNA chip
1998-2016	Total	51.3% (20/39)	HR	18 (65.0%; 13/20), 16 (15.0%; 3/20), 31 (10.0%; 2/20), 35 (5.0%; 1/20), 39 (5.0%; 1/20), 68 (5.0%; 1/20)	
			LR	32 (10.0%; 2/20), 6 (5.0%; 1/20)	

**Abbreviations:** HPV: human papillomavirus, HR: high-risk, ISH: in situ hybridization, LR: low-risk, PCR: polymerase chain reaction, PCR-RFLP: polymerase chain reaction-restriction fragment length pleomorphism assay;

* The present study

In the present study, all the five cases we examined expressed block p16 positivity, which was consistent with the presence of high-risk HPV. In addition, focal ER and PgR positivity in 1 case was consistent with a previous study that detected ER (2 cases) and PgR (1 case) among 11 cases of GCC [16]. However, considering that the remaining 4 cases were negative for both ER and PgR, and most cervical carcinomas does not express these hormone receptors, their expression may be unusual in GCC.

Cytological differential diagnoses of cervical GCC include atypical reparative cells, nonkeratinizing squamous cell carcinoma with severe inflammation, lymphoepithelioma-like carcinoma, and malignant melanoma. Pak et al. [17] reported a false-negative rate of 33% when performing a cytological diagnosis of GCC. In GCC, frequently appearing syncytial tumor cell arrangements and abundant cytoplasm may deceive cytopathologists. In typical repair, cells occur primarily in monolayer sheets and syncytia and contain prominent nucleoli. However, nuclear piling, significant anisonucleosis, and irregularities in chromatin distribution that exceed changes in typical repair, can result in the false exclusion of carcinoma. Nevertheless, atypical reparative reactions lack both tumor diathesis and nuclear hyperchromasia. Moreover, isolated atypical tumor cells in reparative reactions are uncommon [13]. Compared with nonkeratinizing squamous cell carcinoma with inflammation, GCC tumor cells are larger and more polygonal with larger nuclei and distinct cell membranes. Nonkeratinizing squamous cell carcinoma has more oval nuclei with coarser chromatin, which is frequent distributed along the nuclear membrane. In lymphoepithelial-like carcinoma, cell membranes are indistinct, and the nuclear chromatin is distributed peripherally, marginating the nuclear membrane. Although eosinophils are often observed in GCC, in lymphoepithelial-like carcinoma the majority of infiltrating inflammatory cells are lymphocytes and plasma cells, which might be useful for differentiation. However, it is not clear if there is a significant difference in proportion of the inflammatory cells between these 2 diseases because in the present cases, lymphoplasmacytic infiltrates were dominant. A misdiagnosis of malignant melanoma might be avoided by the absence of cytoplasmic melanin pigments and epithelial marker immunoreactivity. GCC neither expresses HMB-45 nor Melan A.

In conclusion, we have described the cytomorphology, histopathology, immunophenotype, and HPV genotypes of cervical GCC. We demonstrated that distinct cytoplasmic margins are a major cytomorphological and histopathological feature of GCC. Cytoplasmic molding and intercellular window formation might be useful cytomorphological characteristics for a differential diagnosis of GCC, especially in liquid-based preparations. In addition, high-risk HPV infection in all cases supported the notion that high-risk HPV is involved in the pathogenesis of GCC.

## MATERIALS AND METHODS

### Case selection

Glassy cell features were defined based on the 3 main criteria originally reported by Cherry and Glucksmann [6] and amplified by Littman et al. [2] as: (1) cells with a moderate amount of ground-glass or finely granular cytoplasm that stains faintly blue with hematoxylin and eosin, (2) distinct cytoplasmic borders that stain with eosin and periodic acid-Schiff, and (3) large nuclei with conspicuous nucleoli. The present study was restricted to cases in which the glassy cell features constituted at least 95% of the specimen. All available slides of 9 poorly differentiated adenosquamous carcinoma cases were reviewed. Five cases of cervical GCCs were included in the present study. Pretreatment cytological specimens were available for all 5 cases. The present study was reviewed and approved by the Institutional Review Board of Severance Hospital, Yonsei University Health System, Seoul, Republic of Korea (2016-1010-001).

### Liquid-based cytological specimen preparation

For liquid-based cytological smears, samples were taken from the fornix, portio, and endocervix. The ThinPrep test (Hologic Inc., Marlborough, MA, USA) was performed using an automated liquid-based monolayer cell preparation system (ThinPrep 2000 system; Hologic Inc.), according to manufacturer's recommendations. Briefly, the samples were immersed in CytoLyt buffer (Hologic Inc.) and then transferred to a PreservCyt bowl (Hologic Inc.). Cells were released by pushing the brush to the bottom, forcing the bristles apart, and swirling the brush in the fluid. A cylinder containing a filtration membrane was placed in the bowl and rotated to ensure homogeneous cell distribution. Erythrocytes and mucus were allowed to penetrate the filtration membrane under negative pressure, leaving the cell membranes on the filtration membrane. Each ThinPrep slide was fixed in ethanol and stained using the Papanicolaou method. Tumor diathesis, cellular composition, and cellular arrangement of the specimens were evaluated.

### Histopathological examination

The biopsied specimens were fixed in 10% neutral-buffered formalin and embedded in paraffin blocks. From each formalin-fixed, paraffin-embedded block, 4-μm sections were cut and stained with hematoxylin and eosin. A variable number of hematoxylin and eosin-stained slides from each case were available for review. Among these, the most representative slide, containing an appropriate volume of tumor and possibly normal cervical tissue, was chosen for immunohistochemical staining and HPV genotyping.

### Immunohistochemistry

The formalin-fixed, paraffin-embedded sections were deparaffinized and rehydrated using xylene and alcohol. Immunohistochemical staining was performed using the Ventana Benchmark XT automated staining system (Ventana Medical Systems, Tucson, AZ, USA) or the Dako Omnis (Dako, Agilent Technologies, Carpinteria, CA, USA), according to the manufacturer's instructions. Antigen retrieval was performed using Cell Conditioning Solution (CC1; Ventana Medical Systems) or EnVision FLEX Target Retrieval Solution, High pH (Dako, Agilent Technologies). Sections were incubated with primary antibodies (Table 3). After chromogenic visualization, using ultraView Universal DAB Detection Kits (Ventana Medical Systems) or EnVision FLEX /HRP (Dako, Agilent Technologies), slides were counterstained with hematoxylin. Appropriate positive and negative controls were stained concurrently to validate the staining method.

**Table 3:** Antibodies used for immunohistochemical staining

Antibody	Source	Clone	Dilution
Carcinoembryonic antigen	Dako, Agilent Technologies Inc., Carpinteria, CA, USA	II-7	1:400
Estrogen receptor	Thermo Fisher Scientific Inc., Fremont, CA, USA	SP1	1:100
Progesterone receptor	Dako, Agilent Technologies Inc., Carpinteria, CA, USA	PgR 636	1:50
p16	Ventana Medical Systems, Tucson, AZ, USA	E6H4	Prediluted
p40	Biocare Medical, Concord, CA, USA	Polyclonal	1:200

### Human papillomavirus genotyping

We performed polymerase chain reaction (PCR)-based microarray for HPV genotyping, using a commercially available HPV 9G DNA chip (BMT HPV 9G DNA Chip; Biometrix Technology, Chuncheon, Republic of Korea) [39]. The 9G test examined the presence of 14 high-risk (16, 18, 31, 33, 35, 39, 45, 51, 52, 56, 58, 59, 66, 68) and 5 low-risk (6, 11, 34, 40, 42) HPV types; analyses were performed according to the manufacturer's instructions [40]. Briefly, the PCR mixture consisted of 10 μL of the extracted target DNA, 10 μL of the primer set (provided by the manufacturer), and PCR premix (provided by the manufacturer), which contained dNTP and Taq DNA polymerase in an amplification buffer. Amplification was performed using the following steps: predenaturation for 5 min at 94°C; 40, 30-s denaturation cycles at 94°C; 40, 30-s annealing cycles at 45°C; 40, 30-s elongation cycles at 72°C; and a final 5-min elongation step at 72°C. PCR products were electrophoresed in a 2% agarose gel to confirm successful amplification. Each hybridization chamber of the 9G was covered with a mixture of the hybridization solution (35 μL) and PCR product (15 μL), followed by incubation at 23–26°C for 30 min. After washing, array images were scanned and imaged using a fluorescent scanner (ScanArray GX Microarray Scanner, PerkinElmer Life and Analytical Sciences, Waltham, MA, USA).
